# Identifying the phonological backbone in the mental lexicon

**DOI:** 10.1371/journal.pone.0287197

**Published:** 2023-06-23

**Authors:** Michael S. Vitevitch, Mary Sale

**Affiliations:** University of Kansas, Lawrence, KS, United States of America; Hong Kong Baptist University, HONG KONG

## Abstract

Previous studies used techniques from network science to identify individual nodes and a set of nodes that were “important” in a network of phonological word-forms from English. In the present study we used a network simplification process—known as the backbone—that removed redundant edges to extract a subnetwork of “important” words from the network of phonological word-forms. The backbone procedure removed 68.5% of the edges in the original network to extract a backbone with a giant component containing 6,211 words. We compared psycholinguistic and network measures of the words in the backbone to the words that did not survive the backbone extraction procedure. Words in the backbone occurred more frequently in the language, were shorter in length, were similar to more phonological neighbors, and were closer to other words than words that did not survive the backbone extraction procedure. Words in the backbone of the phonological network might form a “kernel lexicon”—a small but essential set of words that allows one to communicate in a wide-range of situations—and may provide guidance to clinicians and researchers on which words to focus on to facilitate typical development, or to accelerate rehabilitation efforts. The backbone extraction method may also prove useful in other applications of network science to the speech, language, hearing and cognitive sciences.

## Introduction

**Fig 1:**
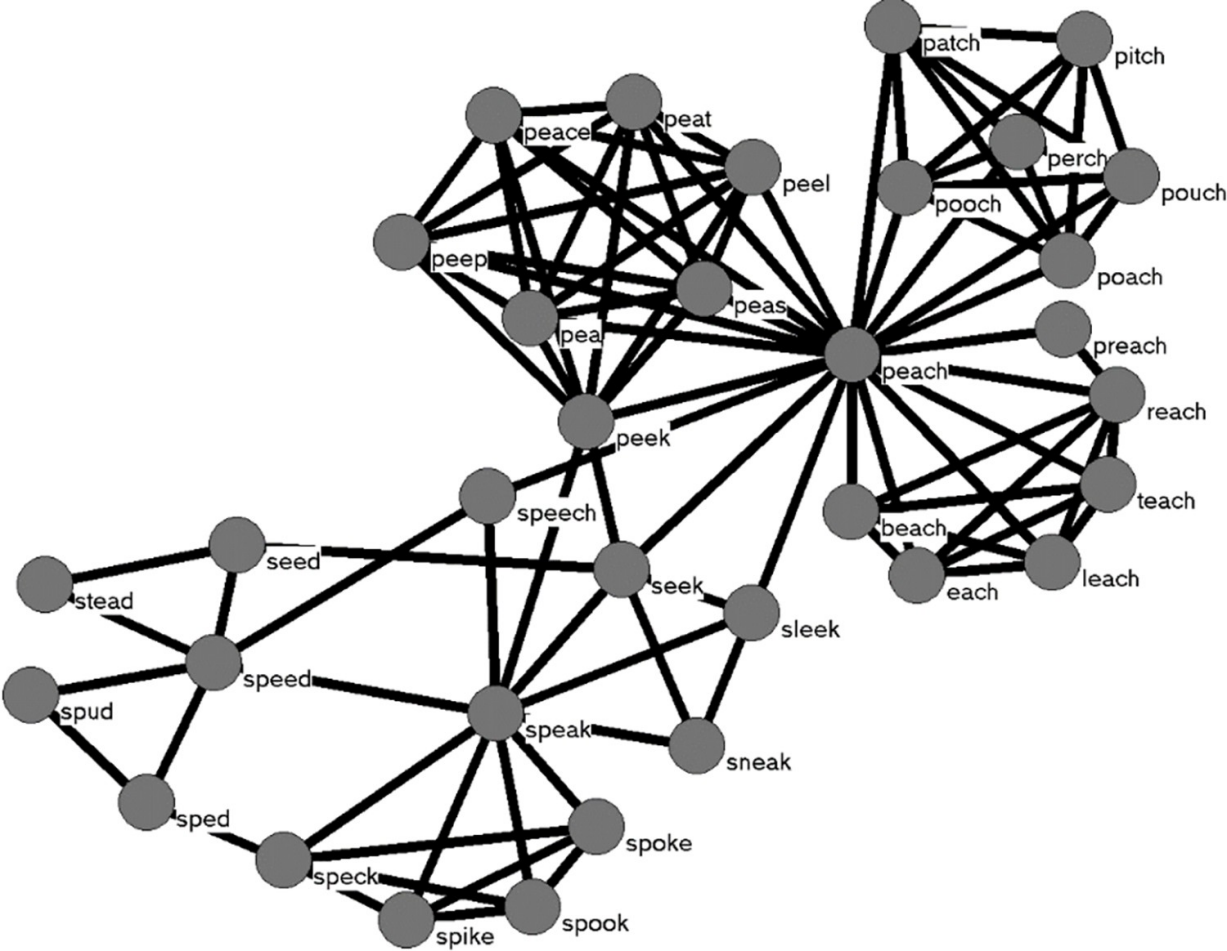
Nodes represent words, and edges are placed between words that sound similar to each other. In this network, phonological similarity is defined by a simple computational metric (add, delete, or substitute a phoneme in a word to form another word), but phonological similarity can be defined in other ways (e.g., [8, 9]).

The mathematical tools of network science are being used increasingly in the speech, language, hearing, and cognitive sciences to better understand typical processing as well as various disorders that affect speech, language, and hearing (e.g., [1–5]). An early application of network science to language mapped the phonological similarity that existed among 19,340 words believed to be stored in the mental lexicon of a typical adult [6, 7]. *Nodes* represented phonological word forms, and *edges* connected words that were phonologically similar based on the addition, deletion, or substitution of a phoneme to form a web-like *network*, a portion of which is shown in Fig 1 (see [8, 9] for other ways to define phonological similarity).

A central tenant of network science is that the structure of the network influences processing in that system [10–12]. Computational analyses of several other languages by [13] found the same structural features in the networks of those languages that were previously observed in the phonological network of English [6], suggesting that the structure of phonological networks is not unique to English. Subsequent psycholinguistic experiments in English found that various measures of the phonological network influenced spoken word recognition [14], speech production [15], word-learning [16], long- and short-term memory [17], and perception of the speech to song illusion [18] in typically-developing language users as well as in people who stutter [19] and in people with aphasia [20].

Among the various measures one can make of individual nodes in a network (i.e., the micro-level), of a subset of nodes in the network (i.e., the meso-level), or of the whole network (i.e., the macro-level), are metrics that allow one to identify nodes that are “important” in the network in some way. Two previous studies used different methods to identify individual nodes and a subset of nodes in the phonological network that were “important” in some way.

In [21] the network science measure known as *closeness centrality* was used to identify “important” nodes in the network. Closeness centrality measures the average distance between a node and all other nodes in the network, and is therefore considered a characteristic of an individual node [22]. In [21] it was found that words like *can* with high closeness centrality (i.e., it is close to many other words in the lexicon) were responded to more quickly in several psycholinguistic tasks than words like *cure* that were similar in several important psycholinguistic variables (e.g., frequency of occurrence, word-length, etc.), but had low closeness centrality (i.e., it is far from other words in the lexicon), demonstrating that a micro-level measure of “importance” can influence processing.

In a different study [23], an algorithm developed by [24] was used to identify a set of “important” nodes in the network called keyplayers. *Keyplayers* are considered “important” because the removal of this set of nodes results in the maximal fracturing of the network. It was found in [23] that the set of words identified as keyplayers were responded to more quickly in several psycholinguistic tasks than words that were similar to the keyplayer words in several important psycholinguistic variables (e.g., frequency of occurrence, word-length, etc.), but which were not in the set of keyplayers. Importantly, being a keyplayer is a characteristic of a set of nodes, not of individual nodes as in the closeness centrality measure used in [21]. Thus, the findings in [23] demonstrate that a meso-level measure of “importance” can influence processing.

In the present study we used another approach—extracting the *backbone*—to identify “important” nodes in the phonological network. In contrast to identifying individual nodes [21] or a set of nodes [23] that are “important” in the network, the backbone approach can be thought of as a whole-network/macro-level approach to identify “important” nodes. In the backbone approach, the essence of a larger, complex network is distilled into a smaller, simplified subnetwork that maintains the basic and crucial features of the original network [25]. The smaller, simplified subnetwork is obtained by discarding redundant or unnecessary edges. Thus, the nodes and edges that appear in the subnetwork that is extracted via the backbone method can be considered a way to identify “important” nodes/edges in the network at the whole-network/macro-level.

One thing that makes the backbone method appealing to use is that often, the smaller, simplified subnetwork reveals relationships that may have been hidden in the larger, more complex network. For example, backbone extraction was used on a network of US Senators who co-sponsored bills to reveal a smaller, simplified subnetwork that provided evidence for partisan polarization in the Senate, which was not evident using other network measures or in the larger, complex network [25]. In the context of a network of phonologically related word-forms, the backbone may capture the distinctive (i.e., marked) phonological features that must be retained to differentiate between phonemes in words, and the redundant edges removed by the backbone procedure may reflect the “default” features of phonemes in words that are easily predicted by phonological rules (and can therefore be discarded) as proposed by various theories of phonological underspecification [26–29].

In the present study we used the backbone package in *R* [25] to extract the backbone of the phonological network first examined in [6], and then compared the lexical and network characteristics of the words in the backbone to the words that were not in the backbone. The “important” nodes and edges that remain in the backbone might point to a set of essential words and phonological relationships (i.e., a kernel vocabulary [30]) that may prove useful to researchers and clinicians working in various areas including language development, second language learning, and aphasia, and which might not have been revealed using other measures from network science or using more traditional measures from psycholinguistics.

## Methods

The phonological network in [6] was a unipartite network that contained 19,340 nodes representing words, and 31,267 undirected edges. Edges connected words if the addition, deletion, or substitution of a single phoneme changed one word into the other. Additional details about the structure of the original phonological network can be found in the results section in the comparisons between the original network and the extracted backbone.

The backbone package for *R* (v2.1.1; [25]) was used to extract the unweighted backbone from the whole network of 19,340 nodes. The backbone of a network is essentially a simplified and smaller subnetwork that is obtained by removing redundant or unnecessary edges (see [25] for a more technical account of the procedure). There are a variety of backbone models that can be used depending on several factors, including whether the edges are weighted or unweighted, whether one is interested in preserving a hidden hub-and-spoke structure or in revealing a hidden community structure, etc. (for guidance see [25, 31, 32]). Because previous work demonstrated the importance of community structure in the intact phonological network of English [33], we wished to maintain and examine further the simplified community structure that might be revealed in the backbone. Therefore, we used the local graph sparsification model (L-spar; [34]) with the following *R* command and parameter settings: sparsify(escore = "jaccard", normalize = "rank", filter = "degree", umst = FALSE.

The escore parameter determines how to score the importance of the edges with the jaccard coefficient being used to assess the similarity between the neighborhoods of the endpoints of each edge (from 0, no overlap, to 1, complete overlap). The normalize parameter determines the method to normalize the edge scores (from 0 to 1) with the rank setting being used to assign the value of 1 to the strongest edge. The filter parameter determines which edges are retained, with degree indicating that the d^s^ most important edges are retained (s = sparsification parameter, ranging from 0 to 1, with 0 leading to the sparsest backbone where only the strongest edge of each node is retained). In order to obtain the sparsest network possible, we selected *s* = 0 as the sparsification parameter, which resulted in 68.5% of the edges being removed (and 0% reduction in the number of connected nodes). We used Gephi (0.9.2; [35]) to measure various structural features of the original and backbone networks. Additional analyses were performed with *JASP* (Version 0.16.3 [36]).

## Results

The original phonological network from [6] was a unipartite network that contained 19,340 nodes representing words, and 31,267 undirected edges. It had a giant component (i.e., the largest cluster of interconnected nodes in the network) of 6,508 nodes and 29,627 edges. There were 10,256 nodes, such as the words *obtuse* or *spinach*, that were not connected to any other word in the network. Unconnected nodes are called isolates in the network science literature, however, in the context of the phonological network they were referred to as “lexical hermits” [6]. The remaining 2,567 words were connected to each other in 1,019 smaller components that were not connected to other smaller components or to the giant component. These components ranged in size from 2 to 53 nodes in a component, and in the context of the phonological network they were referred to as “lexical islands” [6].

After the extraction of the backbone, the 19,340 nodes were connected via 9,843 edges. Table 1 shows various network values for the original (intact) network, and for the network after the backbone had been extracted. The values reported in Table 1 confirm that the phonological network has been significantly “simplified” by the backbone extraction procedure.

**Table 1:** Comparison of network characteristics in the original network and the extracted backbone network.

	Original Network	Backbone Network
**Number of nodes in network**	19,340	19,340
**Number of edges in network**	31,267	9,843
**Number of nodes in GC**	6,508 (34%)	6,211 (32%)
**Number of edges in GC**	29,627 (95%)	7,968 (81%)
**Average Degree in network**	3.23	1.02
**Average Degree in GC**	9.11	2.57
**Network Diameter**	29	47
**Average Shortest Path Length**	6.04	13.34
**Number of connected components**	1,019	1,071
**Size of components (min.-max.)**	2–53 nodes	2–48 nodes
**Number of Isolates**	10,256 (53%)	10,265 (53%)
**Average Clustering Coefficient**	.32	.087
**Number of communities in network**	11,309 (*Q* = .71)	11,386 (*Q* = .89)

Note: GC = giant component.

To determine what enabled some words to “survive” the extraction process and remain in the simplified giant component after the backbone sparcification process, we compared several psycholinguistic characteristics and several network science measures (that have previously been shown to influence language-related processes) of (1) the words in the giant component of the original network (Original GC), (2) the words that remained in the giant component after the backbone was extracted (GC of Backbone), and (3) words that were previously in the giant component of the original network, but that did not make it in to the giant component of the backbone (Orig. GC/Not Bb). For the network science measures, the values of each measure are for the words before the backbone was extracted. To adjust for the unequal sample sizes and unequal variances the Welch correction for independent sample ANOVA (with adjusted degrees of freedom) was used for all comparisons. The Tukey correction was used to adjust for multiple post-hoc comparisons. Table 2 shows the mean (and standard deviation) values for the analyses reported in this section.

**Table 2:** Psycholinguistic and network science characteristics of words that remained in the giant component after the backbone process and of words outside of the giant component.

	Original GC	GC of Backbone	Orig. GC/Not Bb
**Number of nodes**	6,508	6,211	297
**Familiarity**	5.97 (1.45)	5.98 (1.44)	5.80 (1.54)
**Frequency (log**_**10**_ **)**	0.80 (0.80)	0.81 (0.80)	0.64 (0.66)
**Word length (# of phonemes)**	4.06 (0.93)	4.02 (0.92)	4.83 (1.05)
**Degree**	9.11 (8.30)	9.32 (8.34)	4.63 (5.68)
**Clustering Coefficient**	0.28 (0.25)	0.28 (0.24)	0.27 (0.32)
**Closeness Centrality**	0.174 (0.03)	0.175 (0.03)	0.136 (0.04)

Note: Original GC = words in the giant component before the backbone sparcification process. GC of Backbone = words that remained in the giant component after the backbone sparcification process. Orig. GC/Not Bb = words that were in the giant component before the backbone sparcification process, but that did not survive the backbone extraction process and were no longer in the giant component of the extracted backbone. Degree is the network science term for phonological neighborhood density in psycholinguistics. Means (and standard deviation) values are reported. The values for the network science measures are reported for the words before the backbone was extracted.

*Familiarity* was measured on a seven-point scale, with 1 = *don’t know the word* to 7 = *know the word* [37]. There was no difference in familiarity ratings among the three different conditions of words (*F* (2, 801.71) = 1.82, *p* = .16).

*Word frequency* refers to the average occurrence of a word (per million words) in the language [38]. Because word frequency counts are not normally distributed, a log_10_ transformation was used. A significant difference overall was observed among the three conditions of words (*F* (2, 822.15) = 9.10, *p* < .001). Post hoc comparisons revealed that there was no difference between the words in the original GC and the words in the GC of the backbone (*t* (1) = 0.55, *p* = .85). However, the words that were originally in the GC but ended up not in the backbone were significantly different from the words in the original GC (*t* (1) = -3.42, *p* = .002) and the words in the GC of the backbone (*t* (1) = 3.58, *p* = .001), suggesting that words that typically occur less often in the language did not “survive” the backbone extraction process.

*Word length* was measured as the number of phonemes in the word. A significant difference overall was observed among the three conditions of words (*F* (2, 798.36) = 84.77, *p* < .001). Post hoc comparisons revealed that there was no difference between the words in the original GC and the words in the GC of the backbone (*t* (1) = -2.23, *p* = .06). However, the words that were originally in the GC but ended up not in the backbone were significantly different from the words in the original GC (*t* (1) = 13.96, *p* < .001) and the words in the GC of the backbone (*t* (1) = -14.61, *p* = .001), suggesting that longer words did not “survive” the backbone extraction process.

*Degree* refers in network science to the number of nodes that are directly connected to a given node. In psycholinguistic terms this measure in the phonological network is equivalent to *phonological neighborhood density*, or the number of words that are similar to a given word based on the substitution, deletion, or addition of a single phoneme in any position of the target item [8]. For a review of how degree/neighborhood density influences speech perception and production see [39]. A significant difference overall was observed among the three conditions of words (*F* (2, 846.63) = 92.88, *p* < .001). Post hoc comparisons revealed that there was no difference between the words in the original GC and the words in the GC of the backbone (*t* (1) = 1.46, *p* = .31). However, the words that were originally in the GC but ended up not in the backbone were significantly different from the words in the original GC (*t* (1) = -9.11, *p* < .001) and the words in the GC of the backbone (*t* (1) = 9.54, *p* < .001), suggesting that words with fewer phonological neighbors did not “survive” the backbone extraction process.

*Clustering Coefficient* in the phonological network measures the extent to which phonological neighbors are also neighbors of each other. More precisely, the clustering coefficient (*C*) is the ratio of the actual number of edges existing among neighbors of a given word to the number of all possible edges among neighbors if every neighbor was connected. *C* has a range from 0 to 1. When *C* = 0, none of the neighbors of a given node are neighbors of each other. When C = 1, the neighbors are fully interconnected, meaning every neighbor is also a neighbor of all the other neighbors of a given word. This variable has been shown to influence spoken word recognition [14], speech production [15], word-learning [16], long- and short-term memory [17], and perception of the speech to song illusion [18]. There was no difference in the clustering coefficient values among the three different conditions of words (*F* (2, 791.84) = 0.13, *p* = .88).

*Closeness Centrality* measures the average distance from one node to all other nodes in the network (following the shortest path between any two nodes being considered). This variable has been shown to influence language processing in healthy young adults [21], adults who stutter [19], and adults with aphasia [20]. A significant difference overall was observed among the three conditions of words (*F* (2, 789.61) = 117.62, *p* < .001). Post hoc comparisons revealed that each condition was significantly different from the others: Original GC and the words in the GC of the backbone (*t* (1) = 3.14 *p* = .005); Original GC and the words not in the GC of the backbone (*t* (1) = -19.62, *p* < .001); Words in the GC of the backbone and words not in the GC of the backbone (*t* (1) = 20.53, *p* < .001). These results suggest that words that (on average) are farther away from other words (as indicated by the lower normalized inverse measure of the average distance to all other nodes) do not “survive” the backbone extraction process.

Finally, we examined the community structure of words in the giant component before and after the backbone had been extracted [40]. Communities are smaller sub-groups of nodes that tend to be more connected to each other than to nodes found in another community (see [33]). Using the Louvain community detection algorithm, a commonly used community detection algorithm [41–43], we found that before the backbone procedure was executed the giant component contained 26 communities. Modularity, *Q*, is typically used to measure the extent to which clear, well-defined communities are found in a network [44]. For a formal definition of *Q* see [40]. Positive Q values close to the maximum of +1.0 indicate the presence of clear, well-defined communities in the network. The community detection analyses in the giant component before the backbone procedure had *Q* = .68. For the words in the (smaller) giant component that emerged after the backbone procedure was executed, the words were distributed among 60 communities, with *Q* = .87.

**Fig 2:**
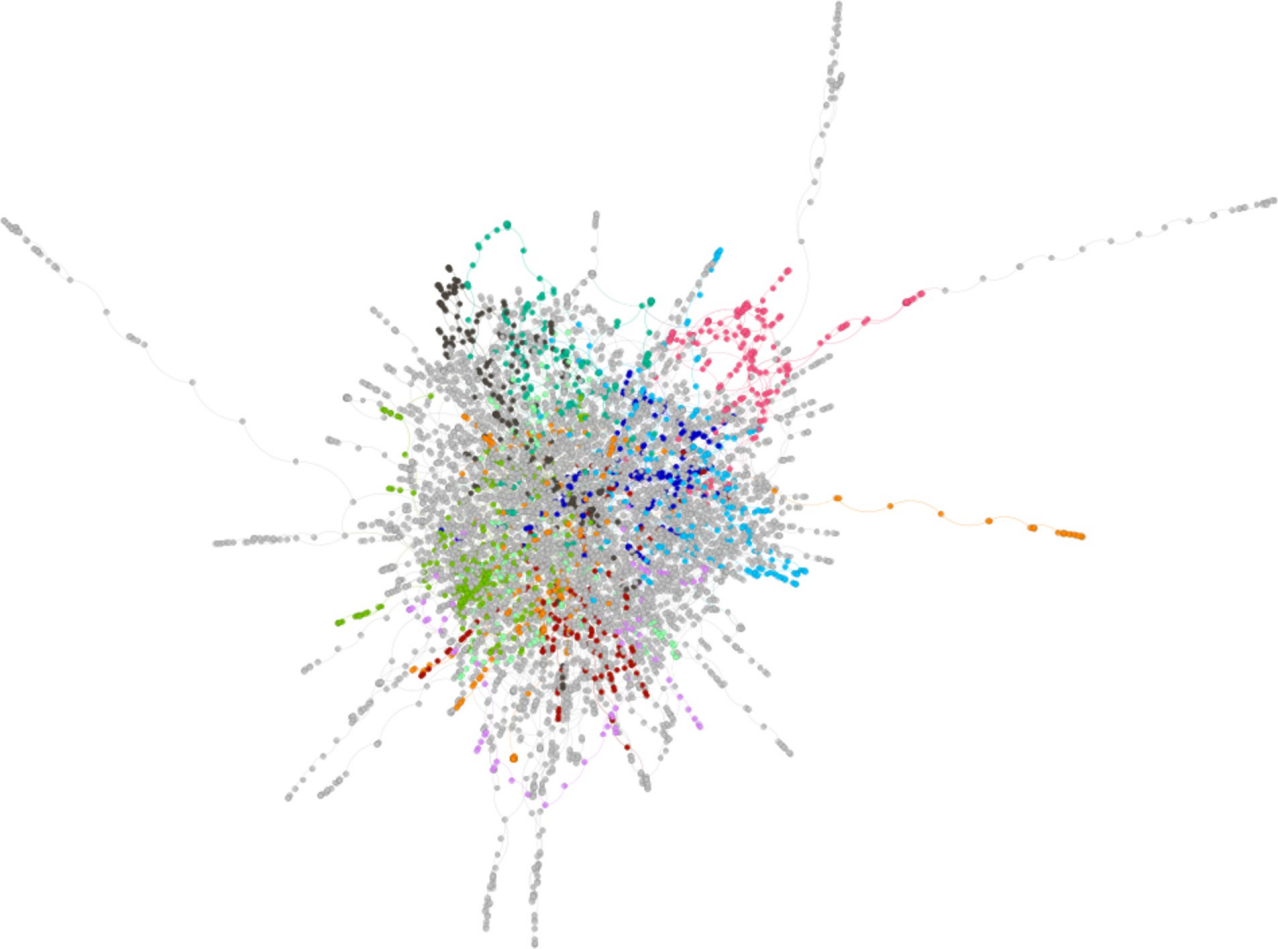
The 60 communities found in the giant component extracted from the backbone procedure. Only the 10 largest communities are colored; all other nodes/communities are grey.

**Fig 3:**
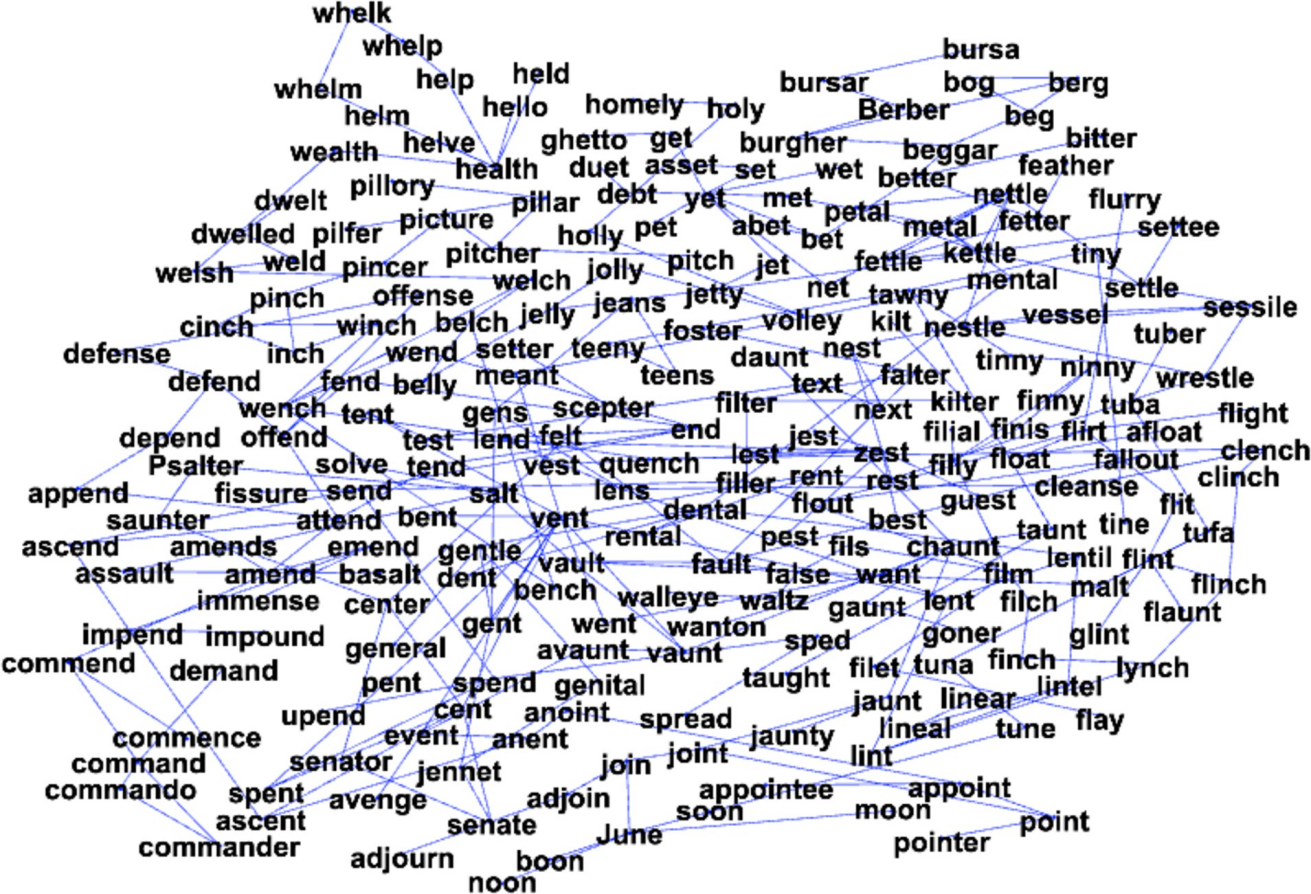
The words found in one of the communities identified in the giant component after the backbone procedure.

Fig 2 shows the 60 communities in the giant component from the backbone (only the 10 largest communities are colored). And Fig 3 shows a single, representative community with words labeling the nodes. Visual inspection of Fig 3 confirms that words in the same community have several phonological sequences in common (e.g., /et/ as in the words *bet*, *debt*, *pet*, *wet*, *set*, etc.), consistent with the initial observation in [33].

## Conclusion

Previous studies have identified at the micro-level individual nodes [21] and at the meso-level a set of nodes [23] in a phonological network that were “important” for lexical processing. In the present study we used a whole-network/macro-level approach to identify “important” nodes. Namely, we extracted the *backbone* from the phonological network of English words. In the backbone approach, a larger, complex network is distilled into a smaller, simplified subnetwork that maintains the basic and crucial features of the original network [25].

The backbone extraction process removed 68.5% of the redundant and unnecessary edges in the phonological network examined in [6]. We compared the psycholinguistic and network measures of the 6,211 words that remained in the giant component (which originally contained 6,508 words) to words were originally in the giant component but were not in the backbone after the sparcification procedure. Words that remained in the giant component of the backbone occurred more frequently in the language, were shorter in length, were similar to more phonological neighbors, and were closer to other words compared to the words that did not “survive” extraction of the backbone. These lexical characteristics suggest that the words in the backbone of the phonological network might form a “kernel lexicon,” or a small but essential set of words that allows one to function (although perhaps not optimally) in a wide-range of situations. Consider the analysis of 4.45 million words extracted from Massive Open Online Courses by [30], who found that the ~5000 most frequent words covered 95% of the course content, and that the ~9000 most frequent words covered 98% of the course content (see also *Up Goer Five* https://xkcd.com/1133/ and *The Thing Explainer* https://xkcd.com/thing-explainer/). Perhaps the words in the phonological backbone constitute a “kernel lexicon” of phonological words-forms that allows a typical speaker to navigate most day-to-day situations.

The edges that remained after the backbone extraction process may reflect important relationships or distinctions between words that cannot be obtained in some other way, such as through phonological rules (e.g., underspecification theories by [26–29]), semantic information, context, or visual features of the lips and jaw in the articulation of the words (as might be used during lip-reading; [45]). Thus, the nodes and edges in the phonological backbone may constitute words and phonological distinctions that are crucial for successful word recognition under less-than-ideal situations, such as when listening to a speaker who is wearing a mask in the era of COVID-19 [46].

Analyses of several network science measures revealed that the removal of redundant edges in the backbone extraction procedure significantly reduced the values for degree, and clustering coefficient, and increased the number of communities, indicating that the network was becoming less interconnected overall. Although the removal of 68.5% of the edges by the backbone extraction procedure significantly reduced the overall connectivity of the network, no words became isolates (i.e., lexical hermits). Rather, any words that were severed from their original structure in the original network formed smaller components (i.e., lexical islands; see [20, 47] for the influence of “lexical islands” on language processing). The fact that the removal of a large percentage of edges resulted in such little damage to the system speaks to the resilience of the phonological network (see also [48]).

Finding a resilient kernel lexicon in the phonological network could be useful for scientists and clinicians in the speech, language, and hearing sciences. The set of words identified in the backbone may provide guidance on which words to focus on to facilitate typical development, and to accelerate rehabilitation efforts. Finally, with the increased application of network science to the speech, language, hearing and cognitive sciences, the backbone extraction method that we explored in the present study may prove useful in other applications of network science. We hope that researchers studying typically developing children (e.g., [3]), children with language disorders (e.g., [1, 2], or the process of reading (e.g., [49] will consider how the various techniques of identifying “important” nodes in a network might be fruitful for advancing those and other research areas.
